# Cyclophosphamide treatment for hypertension and renal injury in an experimental model of systemic lupus erythematosus

**DOI:** 10.14814/phy2.14059

**Published:** 2019-05-23

**Authors:** Victoria L. Wolf, Erin B. Taylor, Michael J. Ryan

**Affiliations:** ^1^ Department of Physiology & Biophysics University of Mississippi Medical Center Jackson Mississippi USA; ^2^ G.V. (Sonny) Montgomery Veterans Affairs Medical Center Jackson Mississippi USA

**Keywords:** Autoimmunity, hypertension, immunosuppression, sex hormones

## Abstract

Cardiovascular disease is the major cause of mortality among patients with the autoimmune disorder systemic lupus erythematosus (SLE). Our laboratory previously reported that immunosuppression with mycophenolate mofetil, a common therapy in patients with SLE, attenuates the development of hypertension in an experimental model of SLE. Cyclophosphamide (CYC) is another common therapy for patients with SLE that has contributed to improved disease management; however, its impact on the development of hypertension associated with SLE is not clear. We tested whether treatment with CYC (25 mg/kg, once/week, IP injection) for 4 weeks would attenuate hypertension in an established female mouse model of SLE with hypertension (30‐week‐old NZBWF1 females). Plasma anti‐dsDNA IgG levels, pathogenic for the disease, were lower in CYC‐treated SLE mice compared to vehicle‐treated SLE mice, suggesting efficacy of the therapy to suppress aberrant immune system function. Mean arterial pressure (MAP) was assessed by carotid artery catheters in conscious mice. Treatment did not attenuate the development of hypertension when compared to vehicle‐treated SLE mice; however, urinary albumin excretion was lower in CYC‐treated animals. Corresponding with the reduction in autoantibodies, data suggest that CYC treatment lowered circulating CD45R^+^ B cells. Paradoxically, circulating CD11b^+^Ly6G^+^ neutrophils were increased in CYC‐treated SLE mice compared to vehicle treated. Estrus cycling data also suggest that CYC treatment had an impact on ovarian function that may be consistent with reduced circulating estrogen levels. Taken together, these data suggest that CYC treatment attenuates autoantibody production and renal disease during SLE, but that the potential to affect MAP may be blunted by the increase in circulating neutrophils and CYC's impact on ovarian function.

## Introduction

Systemic lupus erythematosus (SLE) is a prototypic systemic autoimmune disease with a wide range of clinical manifestations, including a high prevalence of renal involvement and hypertension (Budman and Steinberg 1976; Mandell 1987; Selzer et al. 2001; Al‐Herz et al. 2003; Sabio et al. 2011; Shaharir et al. 2015). Cyclophosphamide (CYC) and mycophenolate mofetil (MMF) are the two drugs commonly used as induction therapy for patients diagnosed with diffuse or moderate to severe focal proliferative lupus nephritis (LN) (Chan 2005). MMF has been shown to reduce blood pressure in both humans and in experimental models of hypertension (Rodriguez‐Iturbe et al. 2002; Herrera et al. 2006; De Miguel et al. 2010; Ferro et al. 2011; Taylor and Ryan 2017a). Our laboratory recently reported that MMF treatment attenuates the development of hypertension in an established, clinically relevant, experimental model of SLE (female NZBWF1 mice) (Taylor and Ryan 2017a). However, the effect of CYC on cardiovascular disease risk factors, such as hypertension, in patients with SLE is not clear.

CYC is a chemotherapeutic agent that inhibits DNA synthesis via the alkylation of nucleic acids, resulting in the miscoding of DNA with subsequent cell destruction (Akawatcharangura et al. 2016). It suppresses both primary cellular and humoral immune responses, but it can also affect any rapidly dividing cells, including gonadal, hematopoietic, and epithelial cells (Akawatcharangura et al. 2016). Therefore, patients taking CYC may experience side effects including hemorrhagic cystitis, bladder cancer, bone marrow suppression, alopecia, and gonadal failure (Akawatcharangura et al. 2016). CYC is typically administered as an induction therapy with either a high‐dose regimen according to NIH protocol or a lower‐dose regimen according to Euro‐lupus protocol (Imran et al. 2016). The high‐dose CYC treatment regimen is 0.5–1 g/m^2^ given intravenously once per month for 6–7 months, and then quarterly following NIH protocol until maintenance therapy is established (Imran et al. 2016). With the low‐dose, Euro‐lupus protocol, CYC treatment regimen is 0.5 g/m^2^ given in six‐biweekly pulses for 10 weeks following by azathioprine treatment (Imran et al. 2016). The Euro‐Lupus trial showed similar 10‐year outcomes in Caucasian patients with mild to moderate LN with the lower‐dose regimen compared to the high‐dose regimen (Houssiau et al. 2002).

Because autoimmunity is associated with prevalent hypertension, and cardiovascular disease is the leading cause of mortality in patients with SLE, it is important to understand the impact of common immunosuppressive therapies on blood pressure (Mody et al. 1994; Abu‐Shakra et al. 1995; Manzi et al. 1997; Bernatsky et al. 2006). In addition, it is now widely established that the immune system plays a central role in the pathogenesis of experimental and human hypertension (Rodriguez‐Iturbe et al. 2017). Therefore, we hypothesized that immunosuppression with CYC would blunt the development of hypertension in an experimental model of SLE. In order to test this hypothesis, we utilized an established experimental mouse model (female NZBWF1 mice) that closely mimics human SLE, including prevalent hypertension. NZBWF1 mice spontaneously develop anti‐double stranded DNA (anti‐dsDNA) autoantibodies, immune complex‐mediated glomerulonephritis, and have contributed significantly to the understanding of human SLE.

## Materials and Methods

### Animals

Female NZW/Lac J (control) and NZBWF1 mice (SLE) (Jackson Laboratories, Bar Harbor, ME) were used in this study. Mice were studied at 30 weeks of age because this typically precedes signs of obvious renal disease (i.e., albuminuria) (Venegas‐Pont et al. 2009). Mice were housed following a 12‐h light/dark cycle in temperature‐controlled rooms and allowed access to chow and water ad libitum. The University of Mississippi Medical Center (UMMC) institutional animal care and use committee approved all experiments that were performed in accordance with the National Institutes of Health Guide for the Care and Use of Laboratory Animals.

### CYC administration

Cyclophosphamide was dissolved in sterile 0.9% saline, and mice were administered 25 mg/kg in 0.1 mL of saline per week by IP injection. Mice not receiving CYC received 0.1 mL 0.9% saline (vehicle). CYC or vehicle was administered once per week for 4 weeks in order to approximate the low‐dose regimen used clinically.

### Blood pressure measurements

Mean arterial pressure (MAP) was measured via catheters implanted in the right carotid artery in conscious, freely moving mice as previously described (Mathis et al. 2011, 2012, 2013, 2014; Venegas‐Pont et al. 2011).

### Renal injury

Urinary albumin was used as a marker of renal injury and was measured weekly by dipstick assay (Albustix; Siemens). At the conclusion of the study, overnight urine samples were used to measured urinary albumin excretion rate (mg/day) by ELISA (Alpha Diagnostic International, San Antonio, TX) as previously described (Mathis et al. 2011, 2012, 2013, 2014). Additional markers of renal injury, kidney injury molecule‐1 (KIM‐1) and neutrophil gelatinase‐associated lipocalin (NGAL), were measured by ELISA (R&D Systems, Minneapolis, MN) as per the manufacturer's instructions. Kidney sections were prepared for glomerulosclerosis scoring with hemotoxylin and eosin staining by Histology Core laboratory services for the Department of Physiology and Biophysics at UMMC, and investigators blinded to the samples scored the sections as previously described by our laboratory (Venegas‐Pont et al. 2009).

### Cell preparation

Whole blood was collected via the retro‐orbital plexus from all animals at the termination of the study. EDTA (20 μL/0.5 mL of blood) was used to prevent coagulation. Plasma was isolated following a centrifugation step (350 × *g* for 5 min) and stored at −80°C for later use. Erythrocyte lysis was performed using 10× volume of 1× lysing buffer (Pharm Lyse™, BD Biosciences, San Jose, CA). Samples were vortexed and incubated away from light for 5 min at room temperature before being centrifuged at 200 × *g* for 5 min. To obtain purified peripheral blood leukocytes, the remaining cell pellets were washed with 1× PBS containing 2% FCS and centrifuged at 350 × *g* for 5 min. Cells were suspended in freezing media (90% FCS, 10% dimethyl sulfoxide) and stored at −80°C for later use. Renal immune cells were prepared for flow cytometry as published previously by our laboratory (Taylor and Ryan 2017a; Taylor et al. 2018). Briefly, 5 mL of Roswell Park Memorial Institute (RPIM) containing 200 U/mL DNase and 10 mg/mL collagenase IV was used to homogenize a single kidney from each mouse with the GentleMACS Octo Dissociator (Miltenyi Biotec) with a user‐defined protocol. The homogenate was filtered with a 70‐μm cell strainer and then washed with 1× PBS containing 2% FCS and 2 mM EDTA. The single‐cell suspension was centrifuged (300 × *g* for 10 min) to acquire a cell pellet, which was then suspended in freezing media and stored at −80°C for later use.

### Flow cytometry

Cryopreserved cells were thawed in a 37°C water bath and washed in 1× PBS containing 2% FCS and 0.9% sodium azide (wash buffer). Cells were resuspended following a centrifugation step (350 × *g* for 5 min), aliquoted into flow cytometry tubes, and placed on ice. Anti‐mouse CD32/CD16 (0.5 μg/sample FcR block; BD Biosciences) was added to cells, and samples were incubated on ice for 5 min. The following antibodies were diluted with wash buffer (50 μL/sample; 1:100 dilution), and placed in a single tube‐containing sample to measure the relative percentages of circulating lymphocyte populations: CD3e‐PE‐Cy7 (clone 145‐2C11), CD4‐FITC (clone GK1.5), CD8‐PerCP‐Cy (clone 53‐6.7), and CD45R‐Alexa Fluor (clone RA3‐6B2) (BD Biosciences). The following antibodies were diluted as stated previously and added to a second single tube‐containing sample to measure the relative percentages of circulating myeloid cell populations: CD11b‐PE‐Cy7 (clone M1/70), Ly6C‐FITC (clone AL‐21), and Ly6G‐PE (clone 1A8) (BD Biosciences). Flow cytometry tubes containing sample and antibodies were shaken/tapped gently to mix and incubated for 30 min in the dark. Cells were washed twice with 2 mL of wash buffer, and the supernatant was decanted after each centrifugation step (350 × *g* for 5 min). Finally, cells were resuspended in 300 μL of wash buffer. Kidney cells were stained with viability stain (ZombieGreen, Biolegend, San Diego, CA) in addition to CD3e‐PE‐Cy7 (clone 145‐2C11), CD4‐FITC (clone GK1.5), CD8‐PerCP‐Cy (clone 53‐6.7), and CD45R‐Alexa Fluor (clone RA3‐6B2) (BD Biosciences). Samples were analyzed on a Gallios (Beckman Coulter) flow cytometer in the UMMC flow cytometry core facility with a total of 100,000 events acquired per sample. Subsequent data were analyzed with Kaluza software (Beckman Coulter).

### Autoantibodies

Anti‐double stranded DNA (anti‐dsDNA) was detected in plasma at the conclusion of the study from 34 weeks of age (SLE mice) or 35 (control mice) using the anti‐dsDNA IgG ELISA (Alpha Diagnostic International) as per the manufacturer's instructions as previously described by our laboratory (Venegas‐Pont et al. 2011; Mathis et al. 2012, 2013, 2014).

### Estrus cycle

The estrus cycle was monitored at 33 weeks of age (SLE mice) and 34 weeks of age (control mice) by vaginal cytology. Smears were allowed to air‐dry after sample collection, stored, and stained with methylene blue (1% aqueous solution) at a later date. Samples were collected over four consecutive days to obtain one complete estrus cycle that could be identified by the presence of phase‐specific nucleated epithelial cells, cornified epithelial cells, or leukocytes. Uterine weight was also measured as a crude marker of estrogen status.

### Statistical analysis

All data are presented as a mean ± SEM based on statistical analyses that were performed using GraphPad Prism 7 (GraphPad Software). Two‐way analysis of variance (ANOVA) was used to determine the effect of treatment (CYC vs. vehicle) or group (SLE vs. control) interactions. One‐way ANOVA was used to determine the significance of individual differences between groups with a Tukey post‐hoc test for multiple comparisons. An unpaired *t* test was used to analyze flow cytometry data to determine the difference between treatments. A D'Agostino and Pearson omnibus test was used to test for normality, and two data sets (Fig. 2B and C) were found to be not normally distributed. Therefore, instead of a one‐way ANOVA to determine the significance of individual differences between groups, a Kruskal–Wallis test was used with Dunn's multiple comparisons test on these two data sets.

## Results

Treatment with CYC significantly reduced spleen weight compared to vehicle (0.09 ± 0.01 [*n* = 15] vs. 0.14 ± 0.01 [*n* = 16], *P* < 0.05) in SLE mice suggesting that it effectively suppressed immune system function, while there was no significant difference between CYC‐ and vehicle‐treated (0.12 ± 0.01 [*n* = 17] vs. 0.12 ± 0.01 [*n* = 19], *P* = 0.99) control mice. CYC treatment did not significantly affect body weight with a decrease of 5.7 ± 1.1% (*n* = 15) compared to a decrease of 4.9 ± 1.1% (*n* = 17) in vehicle‐treated SLE mice (*P* = 0.90), suggesting that the health status of the animal was unaffected by the treatment.

### Impact of CYC on MAP

**Figure 1:**
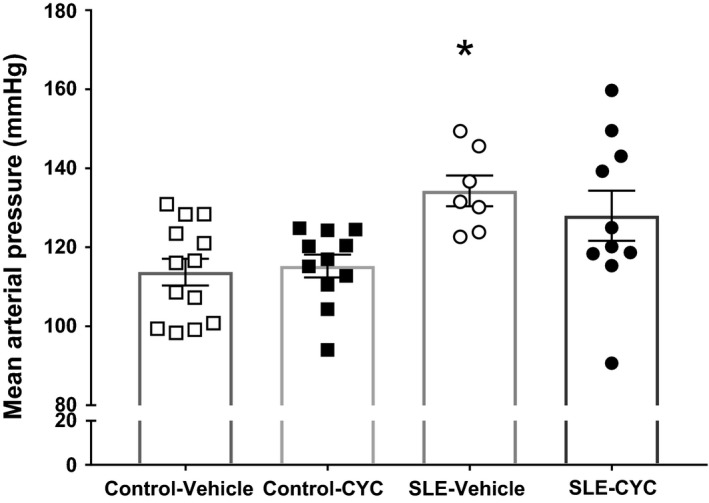
Impact of cyclophosphamide (CYC) on mean arterial pressure (MAP). MAP was measured by indwelling, carotid artery catheter. Vehicle‐treated systemic lupus erythematosus (SLE) mice had increased MAP compared to vehicle‐treated control mice (128 ± 6 vs. 113 ± 3 mmHg, *P < 0.02). Results suggest that blood pressure was not altered by CYC treatment in SLE mice compared to vehicle‐treated SLE mice (128 ± 6 vs. 136 ± 4 mmHg, *P* = 0.6). □ Control Vehicle (*n* = 13), ■ Control CYC (*n* = 11), ○ SLE Vehicle (*n* = 7), and ● SLE CYC (*n* = 10).

To determine the impact of CYC on hypertension in SLE, MAP was measured by indwelling carotid artery catheter in conscious, freely moving mice at the termination of the study in vehicle‐ and CYC‐treated control and SLE mice. As published previously by our laboratory, vehicle‐treated SLE mice had increased MAP compared to vehicle‐treated control mice (128 ± 6 vs. 113 ± 3 mmHg, *P* < 0.05). The results (Fig. 1) show that blood pressure was not altered by CYC treatment in SLE mice compared to vehicle‐treated SLE mice (128 ± 6 vs. 136 ± 4 mmHg, *P* = 0.6). CYC treatment also did not affect blood pressure in control mice (115 ± 3 vs. 113 ± 3 mmHg, *P* = 0.99]. In preliminary studies, long‐term treatment with CYC (8 weeks) did not significantly alter the blood pressure (data not shown).

### Impact of CYC on markers of renal injury

**Figure 2:**
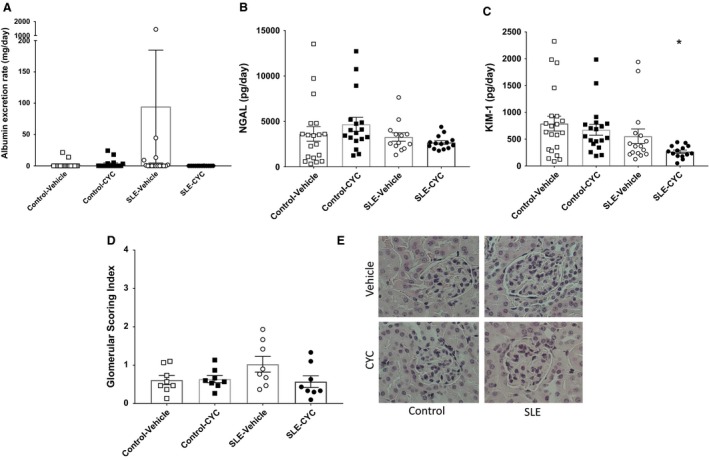
(A) Data suggest that urinary albumin excretion (94.68 ± 90.07 vs. 0.07 ± 0.02 mg/day, *P* = 0.45) is reduced in cyclophosphamide (CYC)‐treated systemic lupus erythematosus (SLE) mice compared to vehicle‐treated SLE mice. No statistical differences were found. □ Control Vehicle (*n* = 21), ■ Control CYC (*n* = 18), ○ SLE Vehicle (*n* = 16), and ● SLE CYC (*n* = 14). (B). Neutrophil gelatinase‐associated lipocalin (NGAL) excretion (pg/day) was not significantly different among vehicle‐ and CYC‐treated control and SLE mice. No statistical differences were found. □ Control Vehicle (*n* = 19), ■ Control CYC (*n* = 17), ○ SLE Vehicle (*n* = 13), and ● SLE CYC (*n* = 14). (C) kidney injury molecule‐1 (KIM‐1) excretion was significantly lower in CYC‐treated SLE mice compared to vehicle‐treated control mice (265.90 ± 30.67 vs. 790.30 ± 139.00, *P < 0.02). □ Control Vehicle (*n* = 21), ■ Control CYC (*n* = 19), ○ SLE Vehicle (*n* = 16), and ● SLE CYC (*n* = 14). (D) Glomerular scoring index was not significantly altered by CYC treatment in control or SLE mice. □ Control Vehicle (*n* = 8), ■ Control CYC (*n* = 8), ○ SLE Vehicle (*n* = 8), and ● SLE CYC (*n* = 8). (E) Renal histological sections were stained with H&E. Representative glomeruli (×40) are from vehicle‐ and CYC‐treated Control and SLE mice.

CYC is commonly prescribed as induction therapy for patients with LN and has previously been shown to effectively prevent the progression of renal disease in experimental models of SLE (Russell et al. 1966; Casey 1968; Russell and Hicks 1968; Gelfand and Steinberg 1972; Hurd 1977; Austin et al. 1986). Urine samples were collected throughout the study, and markers of renal injury, including urinary albumin, KIM‐1, and NGAL excretion rates, were measured at the termination of the study to determine if CYC treatment was renal protective with or without the attenuation of hypertension in an experimental model of autoimmunity. Although CYC treatment does not lower blood pressure, data suggest that mice treated with SLE may be protected against developing albuminuria (urinary albumin excretion 94.68 ± 90.07 vs. 0.07 ± 0.02 mg/day, *P* = 0.45) (Fig. 2A). Therefore, blood pressure changes and albuminuria occur independently of each other in this model. Additional markers of renal injury, urinary NGAL (Fig. 2B) and KIM‐1 (Fig. 2C) were measured to determine if CYC treatment had any other renal protective effects. NGAL excretion (pg/day) was not significantly different among vehicle‐ and CYC‐treated control (3614 ± 798.89 vs. 4672 ± 775.00) and SLE mice (3279 ± 463.10 vs. 2669 ± 201.80). However, KIM‐1 excretion (pg/day) was significantly lower in CYC‐treated SLE mice compared to vehicle‐treated control mice (265.9 ± 30.67 vs. 790.3 ± 139.00, *P* < 0.05). Histological analysis of glomerulosclerosis was performed (Fig. 2D), and SLE vehicle mice had increased glomerulosclerosis; however, CYC‐treated SLE mice trended toward a decrease in glomerulosclerosis compared to vehicle‐treated SLE mice (0.57 ± 0.15 vs. 1.03 ± 0.20; *P* = 0.16). Figure 2E shows representative glomeruli from vehicle‐ and CYC‐treated control and SLE mice.

### Impact of CYC on immune cells and autoantibodies

**Figure 3:**
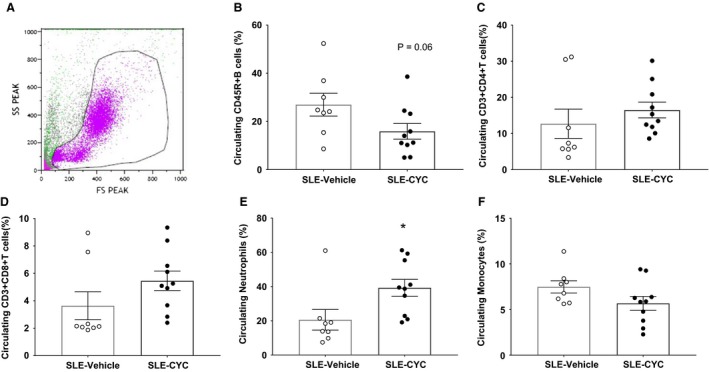
Impact of cyclophosphamide (CYC) on circulating immune cells. Flow cytometry was performed on cryopreserved peripheral blood leukocytes to determine the impact of CYC treatment on lymphoid and myeloid cell populations. (A) Representative scatter plot of peripheral blood leukocytes. (B) Data suggest that CYC treatment reduced CD45R^+^ B cells compared to vehicle treatment in systemic lupus erythematosus (SLE) mice (15.87 ± 8.49% vs. 26.96 ± 4.72%, *P* = 0.06). (C) There was no significant difference in the percentage of circulating CD3^+^CD4^+^T cells between groups or (D) the percentage of CD3^+^CD8^+^T cells.(E) The percentage of circulating neutrophils was significantly increased in CYC‐treated SLE mice compared to vehicle‐treated mice (39.26 ± 4.92 vs. 20.58 ± 6.01, *P < 0.05). (F) There was no significant different in the percentage of circulating monocytes between groups. ○ SLE Vehicle (*n* = 8), and ● SLE CYC (*n* = 10).

**Figure 4:**
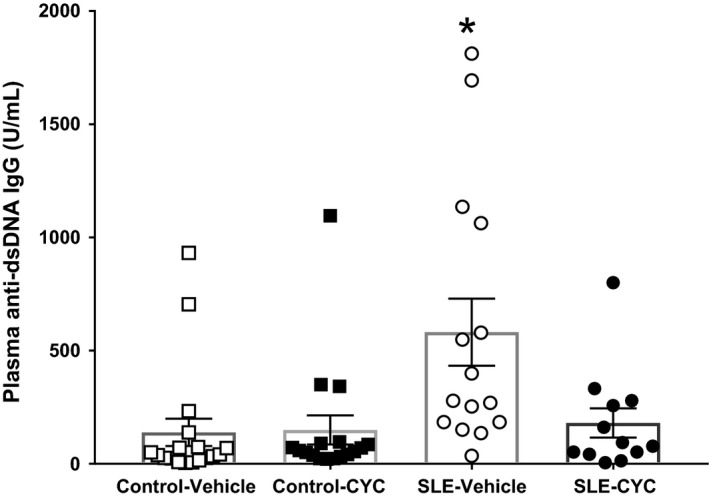
Impact of cyclophosphamide (CYC) on circulating autoantibodies. Systemic lupus erythematosus (SLE) mice treated with CYC had reduced circulating anti‐dsDNA autoantibodies compared to vehicle‐treated SLE mice (180.50 ± 64.68 vs. 581.60 ± 148.3 units/mL, *P < 0.05). Vehicle‐treated SLE mice also had significantly increased plasma anti‐dsDNA IgG (*P < 0.05) compared to vehicle‐ (138.70 ± 60.37 units/mL) and CYC‐treated (149.60 ± 63.86 units/mL) control mice. □ Control Vehicle (*n* = 18), ■ Control CYC (*n* = 17), ○ SLE Vehicle (*n* = 15), and ● SLE CYC (*n* = 12).

**Figure 5:**
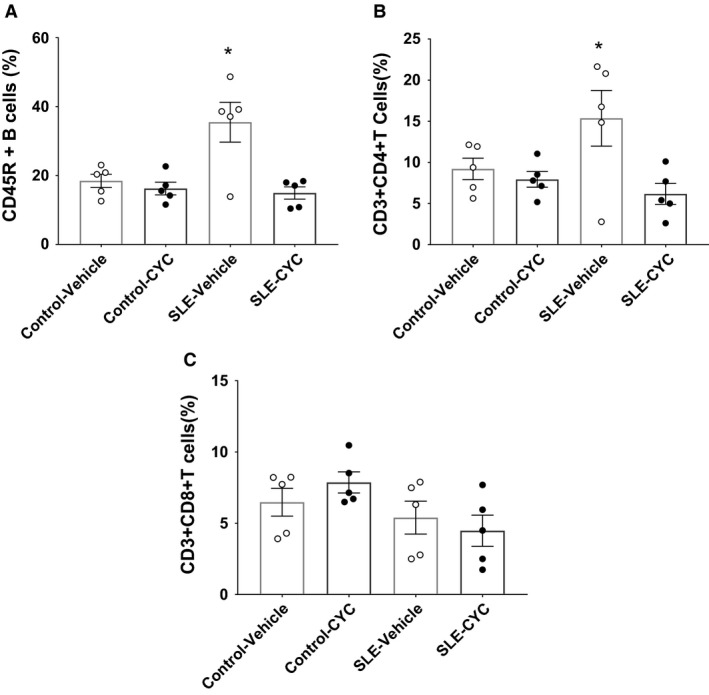
Impact of cyclophosphamide (CYC) on renal lymphocyte infiltration. (A) Renal CD45R^+^ B cells were significantly increased in systemic lupus erythematosus (SLE) vehicle‐treated mice compared to all other treatment groups (*P < 0.05) (B) Renal CD3^+^CD4^+^ T cells were significantly increased in SLE vehicle‐treated mice compared to all other treatment groups (*P < 0.05). (C) Renal CD3^+^CD8^+^ T cells were not significantly different in response to CYC treatment in control or SLE mice. □ Control Vehicle (*n* = 5) ■ Control CYC (*n* = 5), ○ SLE Vehicle (*n* = 5), and ● SLE CYC (*n* = 5).

Flow cytometry was used to determine the impact of CYC on lymphoid and myeloid cell populations in the peripheral blood. CYC treatment reduced circulating B cells (15.87 ± 8.49% vs. 26.96 ± 4.72%, *P* = 0.06) (Fig. 3B) but increased the percentage of circulating neutrophils in SLE mice (39.26 ± 4.92% vs. 20.58 ± 6.01%) (Fig. 3E). Consistent with fewer circulating B cells, SLE mice treated with CYC had reduced circulating anti‐dsDNA autoantibodies compared to vehicle‐treated SLE mice (180.50 ± 64.68 vs. 581.60 ± 148.3 units/mL, *P* < 0.05) (Fig. 4). Analyses of renal immune cells revealed that vehicle‐treated SLE mice had a significantly higher percentage of CD45R^+^ B cells (Fig. 5A) compared to control mice, and CYC treatment SLE mice had significant fewer CD45R^+^ B cells in the kidney compared to SLE vehicle mice (14.94 ± 1.77 vs. 35.48 ± 5.77%, *P* < 0.05). In addition, vehicle‐treated SLE mice had a significantly higher percentage of CD3^+^CD4^+^ T cells compared to all other treatment groups with CYC‐treated SLE mice showing a significant decrease in CD3^+^CD4^+^ T cell (Fig. 5B) infiltration in the kidney compared to vehicle‐treated SLE mice (6.16 ± 1.27 vs. 15.36 ± 3.39%, *P* < 0.05). CYC treatment, however, did not significantly affect CD3^+^CD8^+^ T cell (Fig. 5C) infiltration in control or SLE mice.

### Impact of CYC on estrus cycling

**Figure 6:**
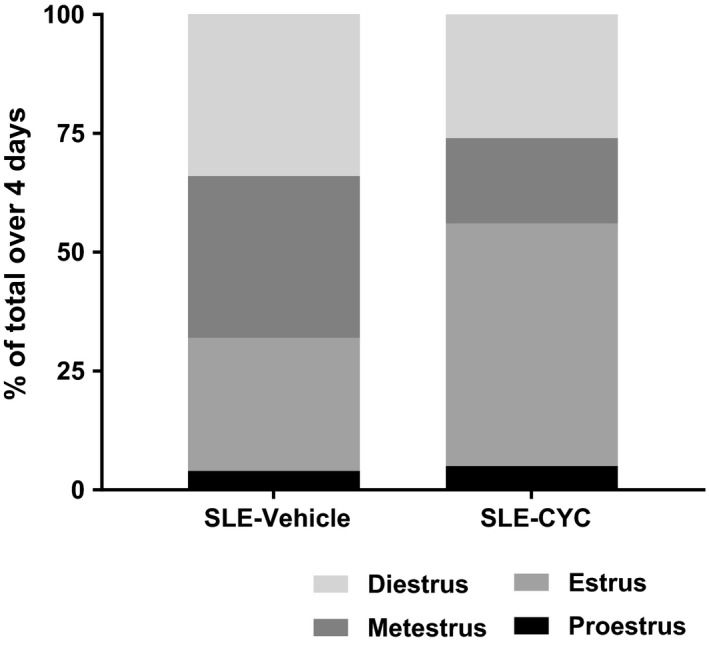
Impact of cyclophosphamide (CYC) on estrus cycling. Data suggest that CYC‐treated systemic lupus erythematosus (SLE) mice (*n* = 15) spent a greater amount of time in the estrus phase of the ovarian cycle compared to vehicle‐treated SLE mice (*n* = 17).

Because CYC has known ovarian cytotoxicity (Akawatcharangura et al. 2016), and loss of estrogen has been shown to exacerbate hypertension in this model (Gilbert et al. 2014), the impact of CYC on estrus cycling was examined. Data (Fig. 6) suggest that the CYC‐treated mice (*n* = 15) spent a greater amount of time in the estrus phase of the ovarian cycle, a finding consistent with impaired ovarian function and altered estrogen status. In addition, uterine weight, which was measured as a crude marker of estrogen status, was not significantly different among vehicle and CYC‐treated SLE (0.08 ± 0.01 vs. 0.07 ± 0.01 g) and control (0.09 ± 0.01 vs. 0.10 ± 0.01 g) mice.

## Discussion

Patients with autoimmune diseases like rheumatoid arthritis or SLE have a significantly increased risk of developing hypertension. We previously demonstrated a direct causal link between autoimmunity and the pathogenesis of hypertension (Mathis et al. 2014; Taylor et al. 2018), thus adding to the overall understanding of the role immune system has in hypertensive disorders (Rodriguez‐Iturbe 2016; Taylor and Ryan 2017b). The purpose of the present study was to test whether CYC, another commonly prescribed chemotherapy for induction therapy in LN, attenuates hypertension during SLE. The major new findings of this study are: (1) different from MMF, CYC does not attenuate the development of hypertension during SLE; (2) CYC treatment reduces markers of renal injury independently of changes in blood pressure; (3) immunosuppression with CYC resulted in an increased relative number of circulating neutrophils; (4) Renal B and T cell infiltration is lower in CYC‐treated SLE mice; (5) chronic treatment with CYC alters estrus cycling in female mice with SLE.

The prevalence of hypertension is increased in patients with SLE typically ranging between 40% and 74% depending on the cohort, compared to only 8–10% in healthy age‐matched women (Budman and Steinberg 1976; Al‐Herz et al. 2003). Using female NZBWF1 mice, an established clinically relevant model of SLE, we previously demonstrated an important role for impaired renal hemodynamic function (Venegas‐Pont et al. 2011), inflammatory cytokines (Venegas‐Pont et al. 2010), reactive oxygen species (ROS) (Mathis et al. 2012), T cells (Mathis et al. 2017), plasma cells, and the associated immunoglobulins (Taylor et al. 2018) in the development of hypertension during SLE. This body of work points to autoimmunity as a driving mechanism for the development of hypertension, a concept that is further supported by clinical studies showing that patients with primary hypertension have increased circulating autoantibodies (Ebringer and Doyle 1970; Suryaprabha et al. 1984; Hilme et al. 1989).

Small clinical studies show that MMF can effectively lower blood pressure in patients with rheumatoid arthritis or psoriasis (Herrera et al. 2006), and we recently showed that MMF attenuates hypertension in female NZBWF1 mice (Taylor and Ryan 2017a). However, the impact of CYC on blood pressure control in patients with SLE is not clear. Understanding the impact of CYC on blood pressure in humans is further complicated by the fact that CYC is often administered concurrently with corticosteroids, which have known blood volume/pressure altering effects (Treadwell et al. 1964). The present study directly assessed whether immunosuppression with CYC lowers blood pressure during SLE. Contrary to the hypothesis, CYC treatment did not attenuate the development of hypertension in mice with SLE. This differs from other studies using CYC in experimental models of hypertension. As an example, CYC reduced blood pressure in Okamoto spontaneously hypertensive rats (SHRs), as measured by tail‐plethysmography (Khraibi et al. 1984). The disparate effects of CYC between these studies may be attributable to key differences in the study design, including both species (mouse vs. rat) and sex (female mice in the present study, male SHR rats). In addition, immunosuppression with CYC in male genetically hypertensive rats of the Lyon strain delayed the onset and attenuated the full development of hypertension while it had no effect on systolic blood pressure in male normotensive control rats (Bataillard et al. 1989). Nevertheless, different from other immunosuppressive therapies, CYC treatment in female mice with SLE does not impact the development of hypertension by as yet to be determined mechanisms.

Because CYC is a commonly used induction therapy for patients with LN, and female SLE mice develop immune complex‐mediated glomerulonephritis, we examined the impact of CYC on markers of renal injury. Previous studies showed that when administered to NZBWF1 mice at the onset of renal disease, CYC treatment attenuates the progression of uremia and proteinuria and prolongs survival (Russell et al. 1966). In this case, however, the incidence of uremia and proteinuria, as well as the degree of kidney damage observed, remained unchanged from baseline measurements, suggesting that CYC did not reverse existing renal disease (Russell et al. 1966; Russell and Hicks 1968). Consistent with these findings, CYC treatment in the present study appeared to protect mice with SLE from developing albuminuria.

KIM‐1 and NGAL were two other urinary markers of renal injury that were assessed at the conclusion of the study. Basal expression of KIM‐1 is typically low in the normal kidney; however, it can be upregulated after ischemia‐reperfusion injury (Ichimura et al. 1998; Alge and Arthur 2015). KIM‐1 protein has been localized to proliferating dedifferentiated epithelial cells of the proximal tubule within 48 h after injury (Ichimura et al. 1998). An increase in urinary KIM‐1 can be observed after renal ischemic or toxic injury (Han et al. 2002; Ichimura et al. 2004). Although KIM‐1 was not increased in mice with SLE in the present study, its excretion was significantly lower in CYC‐treated SLE mice compared to vehicle‐treated control mice. These data suggest that CYC treatment may have preserved proximal tubular structure in SLE mice. The finding that KIM‐1 excretion was significantly higher in vehicle‐treated control mice compared to CYC‐treated SLE mice was surprising. However, one study examined inter‐strain differences in KIM‐1 expression in mouse kidneys suggesting that KIM‐1‐positive proximal tubules are generally increased in the NZW strain compared to other strains (Yoo et al. 2015). Intrarenal NGAL is typically upregulated in response to ischemic or nephrotoxic kidney injury (Mishra et al. 2003, 2004; Supavekin et al. 2003). Urinary NGAL can be detected as early as 3 h postinjury, and data suggest that the thick ascending limb and the collecting duct are the primary sites of intrarenal NGAL production (Mishra et al. 2003; Mori et al. 2005). Human studies suggest that urinary NGAL peaks approximately 6 h after injury, suggesting it is an early marker of renal injury (Mishra et al. 2005; Parikh et al. 2011a,b). This could explain why NGAL excretion was not significantly affected by CYC treatment in the present study. Glomerulosclerosis scoring was consistent with the other markers of renal injury that were measured, suggesting that SLE–CYC mice were protected from exacerbation of glomerular injury compared to SLE vehicle mice. SLE vehicle‐treated mice had an increased glomerular scoring index compared to other treatment groups that is consistent with the well‐documented reno‐protective action of CYC treatment in SLE (Hurd and Ziff 1977).

Reduced markers of renal injury without changes in blood pressure are consistent with our previous work and clinical literature. Immune complex glomerulonephritis is estimated to affect approximately 50% of patients with SLE (Guo et al. 2010), but hypertension in SLE has been shown to occur independently of nephritis in some cases (Shaharir et al. 2015). There is also a disconnect in some mouse models of SLE because MRL/lpr and NZBWF1 mice both develop immune complex glomerulonephritis, but only the female NZBWF1 mouse model develops hypertension (Rudofsky et al. 1984). Although glomerular injury may contribute to hypertension, these findings suggest that there are other disease‐related factors at play. Understanding the underlying factors that promote renal hemodynamic changes during SLE could help explain why albuminuria was impacted independently of hypertension in this study.

Because CYC is a nonspecific immunosuppressive therapy, we examined the impact on several markers of immune system function. Our data showing that SLE mice treated with CYC have reduced numbers of circulating B cells are consistent with our data showing that autoantibody production is attenuated after CYC treatment. Recently published data from our laboratory suggest a mechanistic role for autoantibodies in autoimmune disease‐associated hypertension (Mathis et al. 2014; Taylor et al. 2018). For example, B cell depletion with a mouse anti‐CD20 antibody (equivalent of Rituximab) in female NZBWF1 mice significantly reduced CD45R^+^ B cells and dsDNA autoantibodies, and protected against the development of hypertension and renal injury in SLE (Mathis et al. 2014). Moreover, depletion of the plasma cells that produce the majority of serum immunoglobulins, resulted in a significant decrease in total plasma IgG and anti‐dsDNA IgG levels in SLE mice and caused a significant lowering of blood pressure (Taylor et al. 2018). These data suggest that autoantibodies play an important mechanistic role in the prevalent hypertension associated with autoimmune disease. That CYC did not attenuate the hypertension during SLE even with a reduction in B cells and circulating anti‐dsDNA IgG, suggests the possibility that other changes may have occurred during the treatment to offset the potential beneficial immunosuppressive actions of CYC. One notable change that occurred in response to CYC treatment was the relative increase in circulating neutrophils observed in the CYC‐treated animals. Neutrophils have been linked to both autoimmunity and hypertension because of the key role they play in the production of ROS during the inflammatory process (Glennon‐Alty et al. 2018). The impact of ROS on renal function and the development of hypertension has been extensively reviewed elsewhere (Solak et al. 2016), and our laboratory previously published a study testing the impact of combined antioxidant therapy on hypertension in a mouse model of SLE (Mathis et al. 2012). Others have observed that CYC in the absence of steroid treatment is associated with severe lymphopenia and only mild neutropenia and monocytopenia (Dale et al. 1973). Therefore, the general depletion of immune cells, leaving a relatively higher percentage of neutrophils may be mechanistically important to offset the reduction in B cells and autoantibodies. To date, very little is understood about the contributions of neutrophils to autoimmune‐associated hypertension.

Kidney cell flow cytometry data suggest that among the leukocyte cell population in the kidney, SLE–CYC mice had a significantly lower percentage of B cells and T cells in the kidney compared to SLE vehicle mice. The decrease in CD45R^+^ B cells and CD3^+^CD4^+^ T cells in response to CYC treatment in the kidney is consistent with the mechanism of action that was proposed with MMF treatment as previously published by our laboratory (Taylor and Ryan 2017a). Much like MMF, CYC treatment caused a decrease in autoantibodies that could lead to decreased renal immune complex deposition in SLE mice that could be responsible for a reduction in the number of lymphocytes in the kidneys. The decrease in renal B and T lymphocyte infiltration most likely drives a decrease in local inflammation that explains the positive impact of CYC treatment on renal injury (Katsiari et al. 2010; Bethunaickan et al. 2011).

A second factor that could offset the beneficial effects of CYC‐induced B cell and autoantibody depletion is related to known cytotoxic effects of CYC. Specifically, CYC is directly linked with ovarian toxicity. This is potentially relevant for blood pressure control because ovarian toxicity can change the estrogen status of patients with SLE, and we previously showed a protective role for estrogen in SLE‐associated hypertension (Gilbert et al. 2014). Menstrual abnormalities occur in 53–63% of patients with SLE and this correlates with disease activity (Akawatcharangura et al. 2016). In addition, the prevalence of premature ovarian failure (POF), the termination of menstruation due to loss of ovarian follicular hormone production prior to 40 years of age, is higher in patients with SLE compared to the general population (Mayorga et al. 2016). The prevalence ranges from 12–43% in women with SLE compared to only 1% in the general population (Akawatcharangura et al. 2016). Importantly, CYC treatment is thought to be the primary cause of POF in SLE, with 11–54% of reported SLE patients treated with CYC developing POF (Mayorga et al. 2016). The data from the present study suggest that a 4‐week treatment with CYC in female mice with SLE is sufficient to alter the estrus cycle leading to a greater amount of time spent is the phases of the cycle associated with lower levels of estrogen. Therefore, changing estrus cycle function caused by CYC treatment may also contribute to the inefficacy of CYC to attenuate the development of hypertension.

## Conclusions and Perspectives

Over the past 30 years, there has been a marked increase in the survival rates for patients with SLE. However, in that same time, cardiovascular risk is unchanged and remains the greatest cause of mortality for patients with SLE (Kim et al. 2017). The remaining cardiovascular risk in this patient population provides strong rationale for continued investigation into the therapeutic impact of drugs like CYC that is commonly used in patients with SLE, and provides an opportunity to improve interventional strategies for these patients. The data from the present study are important because they demonstrate that therapies commonly used in patients with SLE may have similar immunological effects (i.e., depletion of immune cells, autoantibodies), but can have very different effects on cardiovascular risk factors like hypertension which are the major determinants of mortality in this patient population.

## Conflict of Interest

None declared.

## References

[phy214059-bib-0001] Abu‐Shakra, M. , M. B. Urowitz , D. D. Gladman , and J. Gough . 1995 Mortality studies in systemic lupus erythematosus. Results from a single center. I. Causes of death. J. Rheumatol. 22:1259–1264. 7562755

[phy214059-bib-0002] Akawatcharangura, P. , N. Taechakraichana , and M. Osiri . 2016 Prevalence of premature ovarian failure in systemic lupus erythematosus patients treated with immunosuppressive agents in Thailand. Lupus 25:436–444. 26621134 10.1177/0961203315617539

[phy214059-bib-0003] Alge, J. L. , and J. M. Arthur . 2015 Biomarkers of AKI: a review of mechanistic relevance and potential therapeutic implications. Clin. J. Am. Soc. Nephrol. 10:147–155. 25092601 10.2215/CJN.12191213 PMC4284423

[phy214059-bib-0004] Al‐Herz, A. , S. Ensworth , K. Shojania , and J. M. Esdaile . 2003 Cardiovascular risk factor screening in systemic lupus erythematosus. J. Rheumatol. 30:493–496. 12610807

[phy214059-bib-0005] Austin, H. A., 3rd , J. H. Klippel , J. E. Balow , N. G. le Riche , A. D. Steinberg , P. H. Plotz , et al. 1986 Therapy of lupus nephritis. Controlled trial of prednisone and cytotoxic drugs. N. Engl. J. Med. 314:614–619. 3511372 10.1056/NEJM198603063141004

[phy214059-bib-0006] Bataillard, A. , M. Vincent , J. Sassard , and J. L. Touraine . 1989 Antihypertensive effect of an immunosuppressive agent, cyclophosphamide, in genetically hypertensive rats of the Lyon strain. Int. J. Immunopharmacol. 11:377–384. 2777432 10.1016/0192-0561(89)90084-2

[phy214059-bib-0007] Bernatsky, S. , J. F. Boivin , L. Joseph , S. Manzi , E. Ginzler , D. D. Gladman , et al. 2006 Mortality in systemic lupus erythematosus. Arthritis Rheum. 54:2550–2557. 16868977 10.1002/art.21955

[phy214059-bib-0008] Bethunaickan, R. , C. C. Berthier , M. Ramanujam , R. Sahu , W. Zhang , Y. Sun , et al. 2011 A unique hybrid renal mononuclear phagocyte activation phenotype in murine systemic lupus erythematosus nephritis. J. Immunol. 186:4994–5003. 21411733 10.4049/jimmunol.1003010 PMC3159403

[phy214059-bib-0009] Budman, D. R. , and A. D. Steinberg . 1976 Hypertension and renal disease in systemic lupus erythematosus. Arch. Intern. Med. 136:1003–1007. 962443

[phy214059-bib-0010] Casey, T. P. 1968 Immunosuppression by cyclophosphamide in NZB × NZW mice with lupus nephritis. Blood 32:436–444. 4175452

[phy214059-bib-0011] Chan, T. M. 2005 Lupus nephritis: induction therapy. Lupus 14(Suppl. 1):s27–s32. 15803928 10.1191/0961203305lu2114oa

[phy214059-bib-0012] Dale, D. C. , A. S. Fauci , and S. M. Wolff . 1973 The effect of cyclophosphamide on leukocyte kinetics and susceptibility to infection in patients with Wegener's granulomatosis. Arthritis Rheum. 16:657–664. 4742843 10.1002/art.1780160510

[phy214059-bib-0013] De Miguel, C. , S. Das , H. Lund , and D. L. Mattson . 2010 T lymphocytes mediate hypertension and kidney damage in Dahl salt‐sensitive rats. Am. J. Physiol. Regul. Integr. Comp. Physiol. 298:R1136–R1142. 20147611 10.1152/ajpregu.00298.2009 PMC2853394

[phy214059-bib-0014] Ebringer, A. , and A. E. Doyle . 1970 Raised serum IgG levels in hypertension. BMJ 2:146–148. 4985868 10.1136/bmj.2.5702.146 PMC1700022

[phy214059-bib-0015] Ferro, C. J. , N. C. Edwards , C. Hutchison , P. Cockwell , R. P. Steeds , C. O. Savage , et al. 2011 Does immunosuppressant medication lower blood pressure and arterial stiffness in patients with chronic kidney disease? An observational study. Hypertens. Res. 34:113–119. 20962786 10.1038/hr.2010.193

[phy214059-bib-0016] Gelfand, M. C. , and A. D. Steinberg . 1972 Therapeutic studies in NZB‐W mice. II. Relative efficacy of azathioprine, cyclophosphamide and methylprednisolone. Arthritis Rheum. 15:247–252. 5031609 10.1002/art.1780150305

[phy214059-bib-0017] Gilbert, E. L. , K. W. Mathis , and M. J. Ryan . 2014 17beta‐Estradiol protects against the progression of hypertension during adulthood in a mouse model of systemic lupus erythematosus. Hypertension 63:616–623. 24366082 10.1161/HYPERTENSIONAHA.113.02385 PMC4365871

[phy214059-bib-0018] Glennon‐Alty, L. , A. P. Hackett , E. A. Chapman , and H. L. Wright . 2018 Neutrophils and redox stress in the pathogenesis of autoimmune disease. Free Radic. Biol. Med. 125:25–35. 29605448 10.1016/j.freeradbiomed.2018.03.049

[phy214059-bib-0019] Guo, Q. , X. Lu , L. Miao , M. Wu , S. Lu , and P. Luo . 2010 Analysis of clinical manifestations and pathology of lupus nephritis: a retrospective review of 82 cases. Clin. Rheumatol. 29:1175–1180. 20556453 10.1007/s10067-010-1517-0

[phy214059-bib-0020] Han, W. K. , V. Bailly , R. Abichandani , R. Thadhani , and J. V. Bonventre . 2002 Kidney injury molecule‐1 (KIM‐1): a novel biomarker for human renal proximal tubule injury. Kidney Int. 62:237–244. 12081583 10.1046/j.1523-1755.2002.00433.x

[phy214059-bib-0021] Herrera, J. , A. Ferrebuz , E. G. MacGregor , and B. Rodriguez‐Iturbe . 2006 Mycophenolate mofetil treatment improves hypertension in patients with psoriasis and rheumatoid arthritis. J. Am. Soc. Nephrol. 17:S218–S225. 17130265 10.1681/ASN.2006080918

[phy214059-bib-0022] Hilme, E. , H. Herlitz , T. Soderstrom , and L. Hansson . 1989 Increased secretion of immunoglobulins in malignant hypertension. J. Hypertens. 7:91–95. 2647845

[phy214059-bib-0023] Houssiau, F. A. , C. Vasconcelos , D. D'Cruz , G. D. Sebastiani , E. D. R. Garrido , M. G. Danieli , et al. 2002 Immunosuppressive therapy in lupus nephritis: the Euro‐Lupus Nephritis Trial, a randomized trial of low‐dose versus high‐dose intravenous cyclophosphamide. Arthritis Rheum. 46:2121–2131. 12209517 10.1002/art.10461

[phy214059-bib-0024] Hurd, E. R. 1977 Effect of cyclophosphamide on interstitial nephritis and tubule cell proliferation in NZB/NZW mice. J. Immunol. 119:1552–1555. 915267

[phy214059-bib-0025] Hurd, E. R. , and M. Ziff . 1977 The mechanism of action of cyclophosphamide on the nephritis of (NZB × NZW)F1 hybrid mice. Clin. Exp. Immunol. 29:132–139. 302170 PMC1541054

[phy214059-bib-0026] Ichimura, T. , J. V. Bonventre , V. Bailly , H. Wei , C. A. Hession , R. L. Cate , et al. 1998 Kidney injury molecule‐1 (KIM‐1), a putative epithelial cell adhesion molecule containing a novel immunoglobulin domain, is up‐regulated in renal cells after injury. J. Biol. Chem. 273:4135–4142. 9461608 10.1074/jbc.273.7.4135

[phy214059-bib-0027] Ichimura, T. , C. C. Hung , S. A. Yang , J. L. Stevens , and J. V. Bonventre . 2004 Kidney injury molecule‐1: a tissue and urinary biomarker for nephrotoxicant‐induced renal injury. Am. J. Physiol. Renal Physiol. 286:F552–F563. 14600030 10.1152/ajprenal.00285.2002

[phy214059-bib-0028] Imran, T. F. , F. Yick , S. Verma , C. Estiverne , C. Ogbonnaya‐Odor , S. Thiruvarudsothy , et al. 2016 Lupus nephritis: an update. Clin. Exp. Nephrol. 20:1–13. 26471017 10.1007/s10157-015-1179-y

[phy214059-bib-0029] Katsiari, C. G. , S. N. Liossis , and P. P. Sfikakis . 2010 The pathophysiologic role of monocytes and macrophages in systemic lupus erythematosus: a reappraisal. Semin. Arthritis Rheum. 39:491–503. 19147182 10.1016/j.semarthrit.2008.11.002

[phy214059-bib-0030] Khraibi, A. A. , R. A. Jr. Norman , and D. J. Dzielak . 1984 Chronic immunosuppression attenuates hypertension in Okamoto spontaneously hypertensive rats. Am. J. Physiol. 247:H722–H726. 6496753 10.1152/ajpheart.1984.247.5.H722

[phy214059-bib-0031] Kim, C. H. , S. G. Al‐Kindi , B. Jandali , A. D. Askari , M. Zacharias , and G. H. Oliveira . 2017 Incidence and risk of heart failure in systemic lupus erythematosus. Heart 103:227–233. 27613169 10.1136/heartjnl-2016-309561

[phy214059-bib-0032] Mandell, B. F. 1987 Cardiovascular involvement in systemic lupus erythematosus. Semin. Arthritis Rheum. 17:126–141. 3334284 10.1016/0049-0172(87)90035-7

[phy214059-bib-0033] Manzi, S. , E. N. Meilahn , J. E. Rairie , C. G. Conte , T. A. Jr. Medsger , L. Jansen‐McWilliams , et al. 1997 Age‐specific incidence rates of myocardial infarction and angina in women with systemic lupus erythematosus: comparison with the Framingham Study. Am. J. Epidemiol. 145:408–415. 9048514 10.1093/oxfordjournals.aje.a009122

[phy214059-bib-0034] Mathis, K. W. , M. Venegas‐Pont , C. W. Masterson , K. L. Wasson , and M. J. Ryan . 2011 Blood pressure in a hypertensive mouse model of SLE is not salt‐sensitive. Am. J. Physiol. Regul. Integr. Comp. Physiol. 301:R1281–R1285. 21917908 10.1152/ajpregu.00386.2011 PMC3213952

[phy214059-bib-0035] Mathis, K. W. , M. Venegas‐Pont , C. W. Masterson , N. J. Stewart , K. L. Wasson , and M. J. Ryan . 2012 Oxidative stress promotes hypertension and albuminuria during the autoimmune disease systemic lupus erythematosus. Hypertension 59:673–679. 22291449 10.1161/HYPERTENSIONAHA.111.190009 PMC3683846

[phy214059-bib-0036] Mathis, K. W. , M. Venegas‐Pont , E. R. Flynn , J. M. Williams , C. Maric‐Bilkan , T. M. Dwyer , et al. 2013 Hypertension in an experimental model of systemic lupus erythematosus occurs independently of the renal nerves. Am. J. Physiol. Regul. Integr. Comp. Physiol. 305:R711–R719. 23926131 10.1152/ajpregu.00602.2012 PMC3798797

[phy214059-bib-0037] Mathis, K. W. , K. Wallace , E. R. Flynn , C. Maric‐Bilkan , B. LaMarca , and M. J. Ryan . 2014 Preventing autoimmunity protects against the development of hypertension and renal injury. Hypertension 64:792–800. 25024282 10.1161/HYPERTENSIONAHA.114.04006 PMC4353646

[phy214059-bib-0038] Mathis, K. W. , E. B. Taylor , and M. J. Ryan . 2017 Anti‐CD3 antibody therapy attenuates the progression of hypertension in female mice with systemic lupus erythematosus. Pharmacol. Res. 120:252–257. 28400152 10.1016/j.phrs.2017.04.005 PMC5836511

[phy214059-bib-0039] Mayorga, J. , D. Alpizar‐Rodriguez , J. Prieto‐Padilla , J. Romero‐Diaz , and M. C. Cravioto . 2016 Prevalence of premature ovarian failure in patients with systemic lupus erythematosus. Lupus 25:675–683. 26678443 10.1177/0961203315622824

[phy214059-bib-0040] Mishra, J. , Q. Ma , A. Prada , M. Mitsnefes , K. Zahedi , J. Yang , et al. 2003 Identification of neutrophil gelatinase‐associated lipocalin as a novel early urinary biomarker for ischemic renal injury. J. Am. Soc. Nephrol. 14:2534–2543. 14514731 10.1097/01.asn.0000088027.54400.c6

[phy214059-bib-0041] Mishra, J. , K. Mori , Q. Ma , C. Kelly , J. Barasch , and P. Devarajan . 2004 Neutrophil gelatinase‐associated lipocalin: a novel early urinary biomarker for cisplatin nephrotoxicity. Am. J. Nephrol. 24:307–315. 15148457 10.1159/000078452

[phy214059-bib-0042] Mishra, J. , C. Dent , R. Tarabishi , M. M. Mitsnefes , Q. Ma , C. Kelly , et al. 2005 Neutrophil gelatinase‐associated lipocalin (NGAL) as a biomarker for acute renal injury after cardiac surgery. Lancet 365:1231–1238. 15811456 10.1016/S0140-6736(05)74811-X

[phy214059-bib-0043] Mody, G. M. , K. B. Parag , B. C. Nathoo , D. J. Pudifin , J. Duursma , and Y. K. Seedat . 1994 High mortality with systemic lupus erythematosus in hospitalized African blacks. Br. J. Rheumatol. 33:1151–1153. 8000745 10.1093/rheumatology/33.12.1151

[phy214059-bib-0044] Mori, K. , H. T. Lee , D. Rapoport , I. R. Drexler , K. Foster , J. Yang , et al. 2005 Endocytic delivery of lipocalin‐siderophore‐iron complex rescues the kidney from ischemia‐reperfusion injury. J. Clin. Invest. 115:610–621. 15711640 10.1172/JCI23056 PMC548316

[phy214059-bib-0045] Parikh, C. R. , S. G. Coca , H. Thiessen‐Philbrook , M. G. Shlipak , J. L. Koyner , Z. Wang , et al. 2011a Postoperative biomarkers predict acute kidney injury and poor outcomes after adult cardiac surgery. J. Am. Soc. Nephrol. 22:1748–1757. 21836143 10.1681/ASN.2010121302 PMC3171945

[phy214059-bib-0046] Parikh, C. R. , P. Devarajan , M. Zappitelli , K. Sint , H. Thiessen‐Philbrook , S. Li , et al. 2011b Postoperative biomarkers predict acute kidney injury and poor outcomes after pediatric cardiac surgery. J. Am. Soc. Nephrol. 22:1737–1747. 21836147 10.1681/ASN.2010111163 PMC3171944

[phy214059-bib-0047] Rodriguez‐Iturbe, B. 2016 Autoimmunity in the pathogenesis of hypertension. Hypertension 67:477–483. 26644240 10.1161/HYPERTENSIONAHA.115.06418

[phy214059-bib-0048] Rodriguez‐Iturbe, B. , Y. Quiroz , M. Nava , L. Bonet , M. Chavez , J. Herrera‐Acosta , et al. 2002 Reduction of renal immune cell infiltration results in blood pressure control in genetically hypertensive rats. Am. J. Physiol. Renal Physiol. 282:F191–F201. 11788432 10.1152/ajprenal.0197.2001

[phy214059-bib-0049] Rodriguez‐Iturbe, B. , H. Pons , and R. J. Johnson . 2017 Role of the immune system in hypertension. Physiol. Rev. 97:1127–1164. 28566539 10.1152/physrev.00031.2016 PMC6151499

[phy214059-bib-0050] Rudofsky, U. H. , R. L. Dilwith , J. B. Roths , D. A. Lawrence , V. E. Kelley , and A. M. Magro . 1984 Differences in the occurrence of hypertension among (NZB × NZW)F1, MRL‐lpr, and BXSB mice with lupus nephritis. Am. J. Pathol. 116:107–114. 6377906 PMC1900373

[phy214059-bib-0051] Russell, P. J. , and J. D. Hicks . 1968 Cyclophosphamide treatment of renal disease in (NZB × NZW) F1 hybrid mice. Lancet 1:440–441. 4169785 10.1016/s0140-6736(68)92778-5

[phy214059-bib-0052] Russell, P. J. , J. D. Hicks , and F. M. Burnet . 1966 Cyclophosphamide treatment of kidney disease in (NZB × NZW) F1 mice. Lancet 1:1280–1284. 4160875

[phy214059-bib-0053] Sabio, J. M. , J. A. Vargas‐Hitos , N. Navarrete‐Navarrete , J. D. Mediavilla , J. Jimenez‐Jaimez , A. Diaz‐Chamorro , et al. 2011 Prevalence of and factors associated with hypertension in young and old women with systemic lupus erythematosus. J. Rheumatol. 38:1026–1032. 21406497 10.3899/jrheum.101132

[phy214059-bib-0054] Selzer, F. , K. Sutton‐Tyrrell , S. Fitzgerald , R. Tracy , L. Kuller , and S. Manzi . 2001 Vascular stiffness in women with systemic lupus erythematosus. Hypertension 37:1075–1082. 11304506 10.1161/01.hyp.37.4.1075

[phy214059-bib-0055] Shaharir, S. S. , R. Mustafar , R. Mohd , M. S. Mohd Said , and H. A. Gafor . 2015 Persistent hypertension in lupus nephritis and the associated risk factors. Clin. Rheumatol. 34:93–97. 25373448 10.1007/s10067-014-2802-0

[phy214059-bib-0056] Solak, Y. , B. Afsar , N. D. Vaziri , G. Aslan , C. E. Yalcin , A. Covic , et al. 2016 Hypertension as an autoimmune and inflammatory disease. Hypertens. Res. 39:567–573. 27053010 10.1038/hr.2016.35

[phy214059-bib-0057] Supavekin, S. , W. Zhang , R. Kucherlapati , F. J. Kaskel , L. C. Moore , and P. Devarajan . 2003 Differential gene expression following early renal ischemia/reperfusion. Kidney Int. 63:1714–1724. 12675847 10.1046/j.1523-1755.2003.00928.x

[phy214059-bib-0058] Suryaprabha, P. , T. Padma , and U. B. Rao . 1984 Increased serum IgG levels in essential hypertension. Immunol. Lett. 8:143–145. 6500633 10.1016/0165-2478(84)90067-1

[phy214059-bib-0059] Taylor, E. B. , and M. J. Ryan . 2017a Immunosuppression with mycophenolate mofetil attenuates hypertension in an experimental model of autoimmune disease. J. Am. Heart Assoc. 6:e005394. 28242635 10.1161/JAHA.116.005394 PMC5524041

[phy214059-bib-0060] Taylor, E. B. , and M. J. Ryan . 2017b Understanding mechanisms of hypertension in systemic lupus erythematosus. Ther. Adv. Cardiovasc. Dis. 11:20–32. 10.1177/1753944716637807 PMC5065379 26985016

[phy214059-bib-0061] Taylor, E. B. , M. T. Barati , D. W. Powell , H. R. Turbeville , and M. J. Ryan . 2018 Plasma cell depletion attenuates hypertension in an experimental model of autoimmune disease. Hypertension 71:719–728. 29378858 10.1161/HYPERTENSIONAHA.117.10473 PMC5843526

[phy214059-bib-0062] Treadwell, B. L. , E. D. Sever , O. Savage , and W. S. Copeman . 1964 Side‐effects of long‐term treatment with corticosteroids and corticotrophin. Lancet 1:1121–1123. 14132625 10.1016/s0140-6736(64)91804-5

[phy214059-bib-0063] Venegas‐Pont, M. , J. C. Sartori‐Valinotti , C. Maric , L. C. Racusen , P. H. Glover , G. R. Jr. McLemore , et al. 2009 Rosiglitazone decreases blood pressure and renal injury in a female mouse model of systemic lupus erythematosus. Am. J. Physiol. Regul. Integr. Comp. Physiol. 296:R1282–R1289. 19193937 10.1152/ajpregu.90992.2008 PMC2698614

[phy214059-bib-0064] Venegas‐Pont, M. , M. B. Manigrasso , S. C. Grifoni , B. B. LaMarca , C. Maric , L. C. Racusen , et al. 2010 Tumor necrosis factor‐alpha antagonist etanercept decreases blood pressure and protects the kidney in a mouse model of systemic lupus erythematosus. Hypertension 56:643–649. 20696988 10.1161/HYPERTENSIONAHA.110.157685 PMC2989495

[phy214059-bib-0065] Venegas‐Pont, M. , K. W. Mathis , R. Iliescu , W. H. Ray , P. H. Glover , and M. J. Ryan . 2011 Blood pressure and renal hemodynamic responses to acute angiotensin II infusion are enhanced in a female mouse model of systemic lupus erythematosus. Am. J. Physiol. Regul. Integr. Comp. Physiol. 301:R1286–R1292. 21900645 10.1152/ajpregu.00079.2011 PMC3213953

[phy214059-bib-0066] Yoo, H. S. , B. U. Bradford , O. Kosyk , T. Uehara , S. Shymonyak , L. B. Collins , et al. 2015 Comparative analysis of the relationship between trichloroethylene metabolism and tissue‐specific toxicity among inbred mouse strains: kidney effects. J. Toxicol. Environ. Health A 78:32–49. 25424545 10.1080/15287394.2015.958418 PMC4281933

